# Overview of the design and development of public health case studies

**DOI:** 10.11604/pamj.supp.2017.27.1.12887

**Published:** 2017-05-28

**Authors:** Ghada N Farhat, Olivia Namusisi, Scott JN McNabb

**Affiliations:** 1 Emory University, Rollins School of Public Health, Atlanta, GA, USA; 2 African Field Epidemiology Network, Kampala, Uganda

**Keywords:** Case study, applied epidemiology, public health competencies

## Editorial

Case studies are an effective tool for simulating real-life public health functions and services [1] in public health training programs. These problem-based learning exercises are enriching and informative to trainees to the extent that they are rooted in the local context and based on country-specific data. In Africa, public health curricula have relied on United States-specific exercises owing to the dearth of analogous examples tailored to African contexts. This supplement introduces 11 new case study exercises based on real events in African contexts and written by experienced Africa-based public health trainers and practitioners. These case studies represent the most up-to-date and context-appropriate case study exercises for African public health training programs. These exercises are designed to reinforce and instil competencies for addressing health threats in the future leaders of public health in Africa. Developing context-specific and culturally tailored case studies has been hampered by the lack of a comprehensive, systematic approach to guide this process. To offset this gap, the Rollins School of Public Health, Emory University – in close collaboration with the African Field Epidemiology Network (AFENET) – developed a training curriculum and implementation plan for the design and development of locally relevant, country-specific public health case studies.

The newly developed methodology – to be published soon – distills best practices and provides a comprehensive, step-by-step approach to case study development. The methodology was piloted in a 2-week course in August 2015 (Case Study Design and Development Course [2] ) delivered to public health professionals and educators from various African universities and Field Epidemiology and Laboratory Training Programs (FELTPs), as well as the U.S. Center for Disease Control and Prevention (CDC). The course, conducted at Emory University, used didactic and hands-on training to develop competency in case study design and development and other pedagogical skills. It guided participants through this step-by-step approach culminating in thoughtful, detailed, locally relevant, culturally tailored, country-specific case studies; they are published here. Course participants facilitated their draft case studies in class and received peer feedback on content and delivery. By the end of the course, participants produced revised case studies ready for use in African FELTPs and public health training programs.

Three cohorts have been trained so far in 2015, 2016, and 2017. A total of 42 participants representing 12 African countries, as well as three participants from the U.S. CDC developed a diverse set of 29 case studies. The inaugural cohort in 2015 produced the 11 case studies published in this special issue of the Pan African Medical Journal. Plans to publish those of the second (2016) and third (2017) cohorts are underway. These case studies and future ones will be accessible by FELTPs across Africa and the world. We trust they will help the next generation of African disease detectives.
